# A process for developing an implementation intervention: QUERI Series

**DOI:** 10.1186/1748-5908-3-17

**Published:** 2008-03-19

**Authors:** Geoffrey M Curran, Snigda Mukherjee, Elise Allee, Richard R Owen

**Affiliations:** 1 Center for Mental Healthcare and Outcomes Research, Central Arkansas Veterans Healthcare System, North Little Rock, Arkansas, USA

## Abstract

**Background:**

This article describes the process used by the authors in developing an implementation intervention to assist VA substance use disorder clinics in adopting guideline-based practices for treating depression. This article is one in a *Series *of articles documenting implementation science frameworks and tools developed by the U.S. Department of Veterans Affairs (VA) Quality Enhancement Research Initiative (QUERI).

**Methods:**

The process involves two steps: 1) diagnosis of site-specific implementation needs, barriers, and facilitators (i.e., formative evaluation); and 2) the use of multi-disciplinary teams of local staff, implementation experts, and clinical experts to interpret diagnostic data and develop site-specific interventions. In the current project, data were collected via observations of program activities and key informant interviews with clinic staff and patients. The assessment investigated a wide range of macro- and micro-level determinants of organizational and provider behavior.

**Conclusion:**

The implementation development process described here is presented as an optional method (or series of steps) to consider when designing a small scale, multi-site implementation study. The process grew from an evidence-based quality improvement strategy developed for – and proven efficacious in – primary care settings. The authors are currently studying the efficacy of the process across a spectrum of specialty care treatment settings.

## Background

In a recent review of diffusion of innovations in health service organizations, Greenhalgh et al. [1] propose that the next generation of research in diffusion should be:

"....participatory: Because of the reciprocal interactions between context and program success, researchers should engage 'on-the-ground' service practitioners as partners in the research process. Locally owned and driven programs produce more useful research questions and data *(e.g., results) *that are more valid for practitioners and policy makers."

Many in the implementation research and organizational change literatures argue that "local participation" in the development of implementation interventions improves their adoption and sustainability [2-7]. Specific models for carrying out such "contextualizing" of best practices, however, are relatively few [1,8]. This article describes a process used by its authors in developing an implementation intervention to assist VA substance use disorder clinics in adopting guideline-based practices for recognizing and treating depression. In short, the *multi-disciplinary participatory process *involved two steps: 1) "diagnosis" of site-specific implementation needs, barriers, and facilitators using key informant interviews and observations of program operations; and 2) use of multi-disciplinary teams of local staff and experts in implementation and clinical issues to interpret diagnostic data, and develop and tailor site-specific interventions.

This process is best considered a method for use in a *diagnostic evaluation *of an implementation intervention. Elsewhere in the *QUERI Series *this process also is referred to as a part of *formative evaluation*. Stetler at al. define formative evaluation as: "A rigorous assessment process designed to identify potential and actual influences on the progress and effectiveness of implementation efforts" [[9], p.S1]. This definition encompasses four evaluative stages that recognize the importance of pre-intervention diagnostic activity, collection of process information during the implementation phase, tracking of goal-related progress, and interpretation of process and outcome data to help clarify the meaning of success, or failure of implementation. Thus, the development process described here is more specifically directed at *pre-intervention *diagnostic activity and the use of resultant data in developing/contextualizing implementation interventions in partnership with local providers, as well as both clinical and research experts. As such, the efficacy of the process should be compared to other models for developing health-focused and/or healthcare system interventions, such as Intervention Mapping [10], Six Sigma [11], Facilitated Process Improvement [12], and Evidence Based Quality Improvement [13]. There is no consensus in the literature regarding an optimal method for developing implementation interventions for healthcare systems, and many have noted the importance of seeking new methods for conducting action-oriented implementation research [6,14-17].

This article is one in a *Series *of articles documenting implementation science frameworks and approaches developed by the U.S. Department of Veterans Affairs (VA) Quality Enhancement Research Initiative (QUERI). QUERI is outlined in Table 1 and described in more detail in previous publications [18,19]. The *Series' *introductory article [20] highlights aspects of QUERI that are related specifically to implementation science, and describes additional types of articles contained in the *QUERI Series*.

**Table 1:** The VA Quality Enhancement Research Initiative (QUERI)

The U.S. Department of Veterans Affairs' (VA) Quality Enhancement Research Initiative (QUERI) was launched in 1998. QUERI was designed to harness VA's health services research expertise and resources in an ongoing system-wide effort to improve the performance of the VA healthcare system and, thus, quality of care for veterans.

QUERI researchers collaborate with VA policy and practice leaders, clinicians, and operations staff to implement appropriate evidence-based practices into routine clinical care. They work within distinct disease- or condition-specific QUERI Centers and utilize a standard six-step process:

1) Identify high-risk/high-volume diseases or problems.
2) Identify best practices.
3) Define existing practice patterns and outcomes across the VA and current variation from best practices.
4) Identify and implement interventions to promote best practices.
5) Document that best practices improve outcomes.
6) Document that outcomes are associated with improved health-related quality of life.

Within Step 4, QUERI implementation efforts generally follow a sequence of four phases to enable the refinement and spread of effective and sustainable implementation programs across multiple VA medical centers and clinics. The phases include:
1) Single site pilot,
2) Small scale, multi-site implementation trial,
3) Large scale, multi-region implementation trial, and
4) System-wide rollout.

Researchers employ additional QUERI frameworks and tools, as highlighted in this *Series*, to enhance achievement of each project's quality improvement and implementation science goals.

### Substance use depression study

This study developed and tested an implementation intervention to assist VA substance use disorder clinics in adopting guideline-based practices for recognizing and treating depression. Within the QUERI process noted in Table 1, this study was directed at Step 4–identifying and implementing interventions to promote best practices. Within Step 4, the study was best characterized as a *small scale, multi-site implementation trial*. Four VA facilities were solicited as participating sites. All were relatively "local" to the lead investigator's team (i.e., within 6 hours drive), and each site had previously been involved in several of the investigators' research and implementation activities. The investigators followed explicit QUERI site recruitment recommendations concerning small scale, multi-site implementation trials, namely that research should be pursued under the "somewhat idealized conditions" of high levels of organization support and commitment for the projects, as well as the ability to leverage established researcher-clinician relationships [20-23]. An outline of the research design and methods is contained in Table 2.

**Table 2:** Methods and key implementation strategies in the substance use depression study

Study Component	Description	Relevant Literature
Purpose	Test a multi-component implementation strategy vs. passive dissemination of evidence materials and implementation tools.	[1, 34–36]
Design	Randomized, quasi-experimental, hybrid design with patient-level clinical outcome data and formative evaluation data collected.	[9, 37]
Sample/programs	Four intensive outpatient SUD treatment programs in southern US, matched on program size/structure and current practices for assessing and treating depression. Two programs randomly selected as intervention sites.	
Evaluation types		
• Diagnostic/developmental (formative evaluation)	Site visits, observations of program operations, key informant interviews with staff, and interviews with veterans with depression in SUD clinics.	[9, 13, 17, 38]
• Implementation-and progress-focused (formative evaluation)	Tracking of: rates of screening, fidelity to screening protocol, consults with program psychiatrists, and use of antidepressants. Frequent phone/e-mail contact with participants to document previously unforeseen barriers/problems and to brainstorm solutions. Number of contacts with site logged.	[9, 38]
• Interpretive (formative evaluation)	Analysis of all formative evaluation data, including key informant interviews at close of implementation period to document stakeholder experiences.	[9, 37–38]
• Summative	Quantitative analysis of patient outcomes. Fifty depression patients from each program surveyed during treatment and at 3- and 6-months post treatment.	[37–38]
Implementation strategy		
• Development Panels	Local development teams made up of clinicians and administrators from each site and the PI considered barrier/facilitator data from development evaluation and literature on depression management implementation strategies/tools. Panel drafted locally-customized clinical care and implementation strategy/tools. Off-site experts consulted to insure that clinical and implementation tools were evidence-based. Panel iteratively redrafted strategy/tools until panel and experts approved of plans.	[1–7, 13, 39–40]
• Other implementation interventions considered by Panel	Clinical reminders, audit and feedback, clinical education, marketing, consumer activation, clinical champions, and multi-component vs. single component interventions.	[1, 3–4, 7, 13, 17, 41–46]
• Facilitation	Internal facilitators to be local "champions" who gather implementation-focused, present at staff meetings, maintain contact with study staff. External facilitation provided by study PI involved problem solving, technical assistance, and creation of educational and clinical support tools.	[9, 17]

In short, the study can be characterized as using a *tiered implementation approach*–gaining acceptance and adoption of depression management practices (depression assessment and initiation of medication) by gaining acceptance and uptake of clinical system changes (i.e., a new screening tool, modified roles for some staff members, a new or enhanced referral mechanism), through the use of selected implementation interventions (i.e., local participation in intervention development, staff education, marketing, and clinic champions).

The intervention development process involves the bolded components in Table 2–namely the **Formative Evaluation **and **Development Panels**. These are the focus of the remainder of the article.

### Approach for developing implementation interventions

#### Rationale

In consultation with implementation experts and through reviews of the relevant theory and empirical research in system and individual change (see citations listed in Table 2), investigators approached the intervention development phase recognizing the need for several factors.

• Theoretical frameworks to guide intervention development and data collection plans.

• A formative evaluation plan that would provide local, diagnostic data to enhance the likelihood of success by leading the team to foreseeable and actionable barriers and facilitators to adoption.

• A partnership with clinical staff for adapting intervention strategies and materials for use in their programs, thereby maximizing the potential fit of the interventions (i.e., "contextualizing") and improving staff buy-in of the clinical and implementation goals.

• Input from clinical and implementation experts so that the study team could bring to the development process credible potential interventions and tools for consideration by local clinicians, and the clinical and implementation interventions developed by investigators and local clinicians remained faithful to their evidence bases.

• Consistent and tangible support from clinic leadership for the intervention.

Investigators chose the PRECEDE model [24,25] to guide intervention development. The acronym stands for "predisposing, reinforcing, and enabling causes in educational diagnosis and evaluation." In the current context, the model led investigators to consider a combination of potential interventions to influence provider behavior: 1) *predispose *providers to be willing to make the desired changes by using interventions such as academic detailing, marketing, or consultation by an opinion leader or clinical expert; 2) *enable *providers to change, for example, by providing screening technologies, clinical reminders, or other clinical support tools; and 3) *reinforce *the implementation of change by providing social or economic reinforcements. This model of healthcare provider change is consistent with several individual-level and organizational change theories, namely the Theory of Planned Behavior [26] (addressing underlying perceptions and beliefs), Social Learning Theory [27] (addressing self-efficacy), and Rogers' model of diffusion [3] (focusing on leadership support and removing barriers to action). These theories were helpful to the investigators in deciding which macro-and micro-level determinants of behavior change would be included in the diagnostic assessments (Table 3).

**Table 3:** Determinants of organizational change assessed by the diagnostic evaluation

Categories of behavior determinants	Examples of specific factors
Characteristics of the external environment	Federal/State regulations, policies, and payment systems. Location (e.g., rural, urban, or geographic or administrative region).
Organizational characteristics	Financial features (e.g., fiscal structure, economic rewards/disincentives). Internal physical environment. Formal organizational features (e.g., staffing; reporting relationships; policies, regulations, rules, and procedures; scope of services; size). Informal organizational features (e.g., culture, norms, social influences, social networks, level of stress, level of burnout, staff tensions).
Characteristics of the clinical practice	Mechanisms for follow-up, referral, and outreach (e.g., support for practice outcomes like continuity, coordination, and access). Mechanisms for enhancing prevention practices. Mechanisms for enhancing disease management practices. Information management mechanisms.
Characteristics of the individual provider	Demographics (e.g., age, sex, ethnicity, recovery status). Education, credentials, (e.g., educational degrees, certification). Ongoing educational experiences (e.g., conferences, lectures, mentored or supervised practice, journal/guideline reading). Knowledge of depressive symptoms/assessment tools/treatments. Skills or competencies (e.g., technical and humanistic). Attitudes, beliefs, potential biases against pharmacotherapy for depression, psychological traits, cognitive processes, readiness to change.
Characteristics of patients	Demographics (age, sex, education, income, employment, ethnicity). Payment mechanisms (e.g., insurance). Severity of substance abuse problems/polysubstance abuse. Extent and severity of comorbid depression/other psychiatric problems. Culture, beliefs, participation, cognitive processes, readiness to take medical advice. Knowledge, skills, information access. Expectations, preferences, adherence. Health and social functioning.
Characteristics of the encounter	Location Type of visit (e.g., scheduled vs. unscheduled). Clinician/patient dyad characteristics (e.g., ethnicity match, sex match).

Note: Modified from Rubenstein et al. [40]

The investigators chose observations of program operations and key informant interviews as methods to gather the necessary diagnostic data. The investigators devised a novel application of evidenced-based quality improvement [13], referred to as "Development Panels," to facilitate the partnership with local clinicians and to provide the process whereby the intervention was customized for optimal consumption. A component of the Panels would be expert feedback on both clinical and implementation interventions that were adapted or developed for the project.

#### Formative evaluation

The following section provides the objectives of each evaluation or development component *and also detailed descriptions of what the investigators actually did in support of them*. The authors intend this section of the article to provide useful and replicable steps.

Data collection in the formative evaluation focused on documenting key influences on the target behaviors or practices (barriers/facilitators), and critical factors affecting the likelihood that the intervention will be implemented and sustained. The assessment investigated a wide range of macro- and micro-level determinants of organizational and provider behavior.

#### Site visit 1

The principal investigator (GC) made an initial visit to the two intervention sites, spending a day reading program policy and procedure manuals and meeting clinical directors. A main objective of this initial visit was to gain information on the programs' current clinical and administrative practices. He first read the manuals, and then met with clinic directors to pose more focused questions concerning clinical policies (especially related to identifying and treating depression). The interviews also were used to gauge the clinical leaders' support of the depression-focused intervention. The program directors had previously provided support letters for the study's grant application, but nine months had passed and the principal investigator wished to assess current attitudes and beliefs. Where necessary, the principal investigator advocated for the adoption of guideline-recommended practices, and came "armed" with brief evidence summaries on depression assessment and antidepressant pharmacotherapy in persons with current substance use disorders. As well, he distributed brief summaries of the VA guidelines concerning the management of depression among persons with co-occurring mood disorders.

The dual role of the principal investigator in these interviews–information gatherer and information giver–was a common dynamic in all interviews during this "diagnostic" phase. The study authors will return to the implications of this dynamic later in the discussion.

#### Site visit 2

Approximately three months later (after completing human subjects safety requirements and necessary local approvals), the principal investigator (GC), co-investigator (SM), and project coordinator (MA) completed the second site visit. They spent three days in each intervention program conducting observations of program operations and key informant interviews.

#### Observations

In the observations, the study team was paying attention to both formal and informal organizational structures: staffing, reporting relationships, policies, norms, leadership and culture, social networks of staff, and staff-patient relationships, to name several (see Jorgensen for a discussion of non-participant observational techniques [28]). The observations themselves were both formal and informal. There were "scheduled" observations, where a study team member or members would witness (with permission) intake interviews, group therapy sessions, educational presentations, or staff meetings. Study teams members also would perform observations in central locations in the programs; for example, the nurses' stations, noting patient flow in the clinic and informal relations among staff and between staff and patients.

A primary goal of the observations was to come away from the site visits with a good understanding of each program's common and accepted ways of doing things, their structures for decision-making, and their favored modes of communication. The team also needed to have a sense of staff cohesion, any staff conflict, and which individual staff members might be experiencing burnout. Based on this information, the study team would then begin to see how the clinical practices to be implemented (i.e., screening, scoring, rapid referrals) might fit into the daily structure of activity, especially including which staff positions or individuals at each site would likely need to be targets of the intervention. The study team met periodically during the site visits to share notes and observations, and the principal investigator compiled the observations after the site visits were over. These data were used (along with data from the key informant interviews) to generate written summaries of program characteristics and pictorial descriptions of clinic processes.

#### Program staff interviews

The study team interviewed 10–14 program staff members at each site during the three-day site visits. Interviewees included program directors, addiction therapists, medical staff, and program administrators. These interviews sought input on: possible in-house barriers/facilitators for the intervention, how the screening might take place, how screening data might be communicated to the clinical team, how the medical director and other clinical staff would be involved and educated as needed in depression management, and how best to educate patients about depression and management techniques such as pharmacotherapy. The interviews also explored potential areas of staff resistance to the intervention, such as negative attitudes toward using antidepressants in this population, and concerns that the program was already too busy to adopt new practices. The interviewers distributed summaries of clinical guidelines and key research findings, and, where appropriate, advocated the positive attributes of the implementation intervention.

The primary goals of the staff interviews were to generate feedback concerning barriers/facilitators to adoption from their perspective – and to gain an overall sense of readiness and willingness for adoption among staff. The study team felt it was important to assure, as much as possible, confidentiality in the interview process. Therefore, an informed consent process was pursued with assurances that the study team would not share participants' specific feedback with their superiors. The study team understood that some barriers to adoption might be due to factions among staff or the behavior/attitudes of certain individuals (including supervisors), and they wanted to maximize the likelihood that staff would feel open enough to share these potentially negative thoughts and feelings. It is important to note here that some information concerning staffing problems, factions, and individual behaviors does not translate into "actionable steps" for interventions. As will be discussed more below, data collection efforts sought to gather as much information as possible about known and suspected indicators of readiness to change. The study team wished to intervene wherever possible to maximize the likelihood of adoption, but barriers that were not addressable were still important to be uncovered and documented, especially in the case of implementation failures.

#### Patient interviews

The study team also interviewed 5–6 veterans who were in substance use disorder treatment and had a history of depression. These interviews sought to document the veterans' treatment history for depression and substance use, to explore the veterans' knowledge of depression and available treatment, and to gauge their thoughts and feelings about pharmacotherapy, undergoing screening and diagnosis, medication adherence, and the process of trying to manage their depression when they are in and out of substance use treatment. Interviewers also sought ideas about what information to include in educational materials for veterans with co-occurring substance use and depressive disorders. The veterans provided informed consent to participate.

#### Data analysis for the formative evaluation

In the spirit of rapid implementation that is central to the QUERI program [21,29,30], the study authors wished to minimize the time from the observations and interviews to the beginning of the Development Panels. The data needed to be analyzed rapidly and prepared for presentation to the Development Panel. Therefore, the study authors chose to pursue a rapid analysis plan similar to that proposed by Sobo et al [31]. The interview tapes would not be transcribed until a later point (to complete the interpretive evaluation and prepare for publications). The interviewers listened to the interviews within two weeks of returning from the site visit and took detailed notes following the structure of the interview guide questions. Within four weeks of returning from the visit, the principal investigator listened to all interviews (including those he did not conduct), compiled all the interview and observational notes, and created barrier/facilitator tables, which were commented on and revised by the other interviewers. These tables summarized and categorized the barriers and facilitators by patient, provider and system levels, and also included "potential solution" and "potential to leverage" columns that contained evidence-based clinical or implementation tools to be considered by the Panel members. An example is provided in Table 4.

**Table 4:** Sample barrier/facilitator table

**Observed/reported barrier**	**Potential solutions**
Poor provider buy-in concerning the importance of recognizing and treating depression.	Facilitated discussion of literature at staff meeting, "rounds" from academic detailer, and provision of guideline synopses.

#### Development Panels

The plan for the Development Panels was informed by the evidence-based quality improvement (EBQI) process as described by Rubenstein et al [13]. The goals of EBQI are to: 1) ensure evidence-based clinical care, 2) tailor the care model to local conditions, 3) minimize clinician time spent on materials or procedure development, and 4) ensure development of local expertise in implementing the care model. EBQI fosters an active researcher-clinician partnership and takes advantage of features known to facilitate innovation, including directly working through the decision-making process regarding intervention design with organizational stakeholders, local adaptation, use of diffusion networks (i.e., opinion leaders), and involvement of researchers as change agents [1,3,7,17,30]. The respective roles for participants in the Development Panels were as follows: researchers were to contribute knowledge about the evidence base for treatment and implementation interventions, as well as materials and tools needed for successful implementation. Clinicians were to contribute local knowledge needed to tailor the evidence-based interventions to meet their own particular needs and to match their organizational capabilities.

The investigators' operationalization of the EBQI model for this project involved:

• Local development teams made up of clinicians and administrators from each site and the study PI,

• Meetings held by conference call over a series of eight weeks,

• Consideration of barrier/facilitator data from observations/interviews,

• Drafting of a locally-customized clinical care and implementation strategy,

• Expert consultation on the clinical care and implementation strategies,

• Iterative re-drafting of the strategies until Development Panel members and experts "approved" the interventions, and

• "Launch date" planning.

The investigators' plan was to complete these tasks in eight weeks time. In reality, the time span from the beginning of the Development Panels to the launch of the intervention at each program was approximately five months. Delays were caused by difficulties in scheduling the multi-stakeholder calls, problems with the installation of an electronic clinical reminder, and difficulties associated with human subjects protection reporting and approvals (common to multi-site projects).

Development Panel candidates were identified in discussions between the principal investigator and the local sponsors of the project. It was hoped that the interviews and observations at the sites would identify promising candidates for the Development Panels and, subsequently, for the roles of "clinical/project champion" during the implementation. The principal investigator used the following criteria to help define a promising candidate: supportive of the clinical goals of the project, energetic and enthusiastic in one's role in the program, respected by other staff members, and willing to take part in the development process (e.g., attend meetings, review draft materials and comment, and take part in presentations to staff). The panel members were not intended to be "opinion leaders" in the classic sense [17]; they were to be "willing and able" participants from varying disciplines within their programs.

Promising candidates were identified and approached for participation in the Panel, and all who were approached agreed to participate. Each panel was made up of a clinical director, a physician, a counselor, and a nurse or other staff member who commonly performed screenings. Before the first meeting, study staff sent the panel members various reading materials: evidence summaries (same as in the interviews), a summary of the study aims, specific instructions on the objectives of the panels, and the barrier/facilitator tables generated for their program. In the meetings, Panel members and the principal investigator discussed ideas for optimal integration of the new clinical practices. As well, they discussed the identified barriers/facilitators from the site visits and discussed potential solutions. As noted above, some barriers were highly addressable with intervention tools, such as the need for brief and valid screener, and some were less so, for example, a complicated intake process in one clinic, where no one staff member saw all incoming patients.

The local customizations of clinical practices to be integrated into routine care and implementation tools developed to support their uptake were designed to match current organization practices and norms. For example, one site already used their electronic medical record system to access brief screening surveys for other conditions and general intake procedures, and they chose to implement the evidenced-based depression screening with an electronic clinical reminder and to order psychiatrist consultations from the reminder through the electronic medical record. The other intervention site preferred paper screeners and either face-to-face or e-mail referrals to a program psychiatrist. The Panel members understood going into the process that such screening and routing of screening data were "mandatory" elements of the clinical intervention, but that the manner in which these practices were carried out were customizable.

There were four experts who consulted on the intervention – two clinical experts in substance use and depression comorbidity and two implementation research experts. The experts consulted via e-mail or phone with the principal investigator exclusively and did not participate in the Panel meetings. This modification to Rubenstein et al.'s EBQI method [13] originated in practical concerns, namely, the difficulty in scheduling meetings with the local staff members and experts, and the principal investigator's desire to allow the experts to provide comments at their convenience. Prior to providing consultation, the experts received written instructions on the scope of their consultative activity. Then they received a written summary prepared by the principal investigator of the locally-customized implementation strategies from each intervention site. The experts reviewed the strategies, provided feedback, and ultimately provided approval.

Once the intervention materials were finalized and all electronic support systems were installed and operational, the Development Panels devised and executed the launch of the intervention at their sites. The Panels chose to use a staff meeting or meetings to introduce the intervention and its tools. The sites chose the date when the intervention began.

#### Intervention components developed and used in implementation

The intervention produced from this process was composed of support tools for the staff and patients, and a group of activities and strategies for both program staff and study staff to support implementation. The tools were developed to facilitate the clinical practices being adopted, namely assessment for non-substance-induced depression and an urgent referral to a program psychiatrist.

The following support tools were developed for the depression management intervention: evidence summaries for staff members concerning depression management, educational materials for patients, a sample depression screener, a computerized clinical reminder to facilitate screening and referral, and template progress note language for medical records. The study team was responsible for developing the tools, making and delivering the necessary number of copies of materials, and supervising the installation and testing of the computerized clinical reminders.

These activities fall under what Stetler [19] refers to as "external facilitation," meaning activities supportive of implementation that are provided by persons external to the clinical setting. External facilitation is itself an implementation intervention that is getting more attention in the literature, and the investigators explicitly included it in the study as an important part of the implementation strategy. However, questions about "what kind of" and "how much" external facilitation to provide, and under what circumstances (i.e., moving from a small- to large-scale implementation project) remain unanswered. The current study measured closely the extent of external facilitation, so as to facilitate analyses of linkages between facilitation and intended clinical change. While detailed descriptions of the devoted resources are beyond the goals of this article, we can state that the principal investigator devoted 16 hours per week, and the project coordinator dedicated 30–40 hours on these facilitation efforts during the development phase of the project.

Formative evaluation-related activities by program staff members in support of effective change also were pursued in the implementation strategy [9]. Two main activities were involved–monitoring of implementation, and devising and implementing changes to the intervention if implementation problems arose. The plan was to have the program staff members who were on the Development Panels do the monitoring and devising. In reality, the monitoring "fell" to one member of the Panel, while the full panel was used to devise solutions to problems. Monitoring implementation involved collecting fidelity measurements, for example, extent of screening, number of referrals made, and the number of referrals successfully completed as well as emerging barriers to implementation.

## Discussion

Site diagnosis and intervention development are not novel exercises. There are numerous tools and models available for consideration, such as EBQI [13], Continuous Quality Improvement [32], Six Sigma [11], and Facilitated Process Improvement [12], to name several. However, there are few guidelines to assist implementation scientists in choosing the implementation methods or interventions best suited for the tasks at hand.

Benedetto [33] recently offered a useful distinction of implementation methods, "evolutionary" versus "revolutionary", based upon the expected degree of organizational or systems change necessary to achieve the desired quality improvement. When a major process redesign is not expected, an evolutionary method of implementation is likely sufficient. For example, a Continuous Quality Improvement-like model could be pursued that involves leadership-authorized, problem-solving teams who create an intervention but do not radically change job descriptions or staffing patterns [32]. Revolutionary methods are those that involve major changes in staffing, funding and culture. These are directed at re-engineering a system, such as Six Sigma [11], where "going back to the drawing board" is possible, and major policies and procedures can be rewritten. Evidence-based quality improvement [13] and its current variant would fall in the class of evolutionary strategies, however, they do involve significant external facilitation, time and effort. If this typology proves valid and useful, perhaps also with distinctive subcategories, the literature will need to validate measures to indicate which type to pursue.

In addition to the variables noted above, a number of additional considerations were taken into account in designing and performing the development process. First, it was directed at the *specific stage of implementation *that was called for in this case–a small scale, multi-site implementation study as defined by QUERI (Phase 2) [20]. A single-site pilot study (Phase 1) had already taken place. In the QUERI framework, these small-scale, multi-site studies are intended to be *efficacy studies *of an organizational intervention. These studies necessitate: rigorous site diagnostic analyses, partnerships with key clinic stakeholders in the intervention development process, significant external facilitation by the study team, and extensive formative evaluations to shape the intervention and both influence and understand its impacts. A future study will prepare the intervention for a large-scale, multi-region implementation trial that should involve significantly less local diagnostic work and external facilitation by study team members (Phase 3).

Next, the substance use disorder treatment programs' relatively low census and small clinical staffs made them good candidates for using key informant interviews, as opposed to survey methods to assess barriers/facilitators to implementation as well as clinic structure and organizational climate. Key informant interviews usually provide a far richer picture than surveys. Because the interviews and site visits could be used additionally for building rapport with the bulk of the clinic staff and for "real time" discussion of concerns and fears, it made pursuing this method even more attractive. Other similarly staffed and organized clinics (HIV clinics for example) could benefit from this qualitative-based combination of site diagnostic activities and marketing. This combination worked well for the current project and saved time in the process (as opposed to performing these talks at different times). There appears to be little discussion in the literature of similar approaches.

Combining data gathering and marketing in the same interview has the potential for creating tension, and it could "backfire" in terms of building rapport. To minimize tension, the interviewers (i.e., the principal investigators or selected co-investigators) would be clear up front about the dual-nature of the interviews. Before an interview started, they would frame it with the staff participants as "a chance to learn more about current practices, hear your thoughts and feelings about them, and for you to provide feedback on some clinical options being considered in the program." These points also were explicit in the informed consent process and forms. During the interview, the interviewers would transition to the "feedback on the clinical practices under consideration" activity by outlining the evidence base in the area and noting the strength of the evidence as a motivator for the program to participate in the project. The interviewers also would state that the program's participation was voluntary. During the feedback section, the interviewers would take very much of a "motivational interviewing" approach to eliciting feedback and ambivalence about adopting new practices. Every barrier raised was affirmed and restated by the interviewers, and ideas for solutions were encouraged. These approaches seemed to help avoid difficult situations; however, more discussion and research is necessary to understand how best to collect diagnostic data from programs and generate positive reactions concerning their involvement in change activities.

Next, the complex pseudo-inpatient nature of many intensive, outpatient substance use treatment programs indicated the usefulness of observations of program operations. As well, these programs vary widely in terms of treatment programming and lengths of stay, so substantial effort is needed to understand the operations of each program. Other such complex clinical care environments also would indicate dedicated observational analyses for which standard methods are available. [28]

Lastly, the investigators sought to keep the Development Panels relatively small in terms of membership, and they wished to use the experts in a consultative manner, as opposed to involving them in the Panels. The process involved three types of stakeholders–external facilitators (principal investigators and study staff), internal facilitators (clinic staff members), and expert consultants. Feasibility was the main concern. The study team tried to keep the time and effort of the internal facilitators and the consultants to a minimum, while meeting the goals of each stakeholder group. Again, these roles were derived specifically for a small-scale, multi-site implementation trial as defined by QUERI, and studies in other phases of roll-out would have different stakeholders and needs.

## Conclusion

The intervention development process described here is presented as a method to consider when designing a small scale, multi-site implementation study. The process grew from an evidence-based quality improvement strategy [13] developed for and proven efficacious in primary care settings. The authors are currently studying the efficacy of the process across a spectrum of specialty care treatment settings, namely VA HIV clinics, VA specialty mental health clinics, and community substance use disorder treatment programs in the United States. Data will be compiled from these efforts to explore the generalizability of the process. In addition, future efforts will translate the process for use in large-scale (regional) roll-outs of evidence-based practices.

In reflecting on this study, the investigators have identified several other important and related areas for research. First, comparisons of survey and key informant interview-derived investigations of organization climate and culture are necessary to determine the most cost-effective ways of collecting this information. Next, future research should determine appropriate sampling techniques for small-scale implementation studies in order to maximize a feasible transition to large scale roll-outs. Also, the crucial elements of effective external facilitation by study staff need to be determined across all phases of implementation research. These gaps in our knowledge, and many others identified in the *QUERI Series *of articles, continue to present barriers to the timely implementation of evidence-based practices.

## Competing interests

The author(s) declare that they have no competing interests.

## Authors' contributions

GC conceived of the study, participated in its design and coordination, and helped draft the manuscript. SM helped conceive the analysis plan of the study, participated in its design and coordination, and conducted interviews and analysis. EA conducted interviews and analysis and conducted literature reviews. RO helped conceive of the study, participated in its design, and provided consultation. All authors read and approved the final manuscript.
